# Supplementary material for: Evidence that dengue virus envelope protein in patient platelets originates from infection of megakaryocytic precursors
Source: PLoS One. 2026 Apr 1;21(4):e0343641. doi: 10.1371/journal.pone.0343641 (PMC13043056; doi:10.1371/journal.pone.0343641)
Supplement: Attachment — Submitted filename: Response to Reviewers.docx [file pone.0343641.s001.docx]

**Response to the Academic Editor**

We sincerely thank the Academic Editor for the careful assessment of our manuscript and for the opportunity to submit a major revision. We fully acknowledge the scope of the reviewers’ concerns and have addressed each comment in detail in the accompanying point-by-point response.

In this revised version, we substantially improved the manuscript by expanding methodological descriptions, clarifying experimental design, strengthening statistical analyses, and revising the presentation and interpretation of results to avoid overstatement. Figures have been reorganized and relabeled where needed, figure legends have been rewritten for clarity, and statistical reporting has been standardized across the manuscript.

For specific points where reviewers requested additional experiments, we carefully evaluated feasibility and have revised the manuscript to ensure that conclusions are fully supported by the available data. In cases where certain experiments could not be added at this stage due to limitations in the availability of specific clinical samples and/or key reagents, we explicitly acknowledged these limitations and clarified the scope of inference in the Discussion, adopting a conservative interpretative approach.

We believe that these extensive revisions address the reviewers’ concerns and significantly strengthen the manuscript. We respectfully submit the revised version for further consideration and appreciate the Editor’s guidance throughout this process.

**Response to Reviewers**

**Manuscript ID**: PONE-D-25-49675

Title: Evidence that intracellular dengue virus envelope protein in patient platelets originates from infection of megakaryocytic precursors

Authors: Alvarado-Silva et al.

Journal: PLOS ONE

We thank the Academic Editor and the reviewers for their careful evaluation of our manuscript and for the constructive comments provided. We have revised the manuscript extensively to address all concerns raised. Below, we provide a point-by-point response to each comment, with changes highlighted in the revised manuscript.

Reviewer #1: In this manuscript, “Evidence that intracellular dengue virus envelope protein in patient platelets originates from infection of megakaryocytic precursors,” the authors investigate whether dengue virus (DENV) infection of megakaryocytic precursor cells leads to the transfer of viral envelope (E) protein to platelets. They use K562 and MEG01 megakaryocytic cell lines, examine differentiation marker CD41, and detect DENV-E protein in platelet-like particles (PLPs) derived from infected cultures. Additionally, they report detection of DENV-E protein in platelets from dengue patients, with a positive correlation to disease severity.
The scientific question is important and clinically relevant. Understanding whether megakaryocytic precursors act as a reservoir for DENV proteins and contribute to platelet dysfunction has implications for dengue pathogenesis. The manuscript presents potentially interesting findings; however, several key aspects of experimental design, methodology, data presentation, and interpretation need to be improved. In its current form, the manuscript contains speculation that is not sufficiently supported by data, and many experiments lack methodological clarity.

We thank the reviewer for this careful assessment. We agree that greater methodological clarity was needed to ensure full transparency and reproducibility. In response, we have revised the Materials and methods section to explicitly describe infection conditions, stimulation timing, normalization strategies, flow cytometry gating, platelet isolation procedures, biological replicates, and statistical analyses. No new experiments were added; rather, previously implicit or dispersed methodological details were consolidated and clarified.

Major Concerns

1-Insufficient methodological detail
Critical experimental parameters are either missing or unclear and needs to be addressed

• The infection protocol for K562 and MEG01 cells needs to be described in detail (MOI calculation, duration, infection conditions).

We thank the reviewer for pointing this out. The infection protocol has now been explicitly detailed in the Materials and Methods section. We have clarified the multiplicity of infection (MOI) used for each cell line, the duration of viral adsorption, washing steps, and post-infection culture conditions to ensure reproducibility and transparency.

• It is unclear when PMA stimulation was performed relative to infection (pre-, post-, or simultaneous).

We agree that this point required clarification. The timing of PMA stimulation relative to viral infection has now been clearly specified. PMA was added simultaneously with viral infection and maintained throughout the culture period as a differentiation control, rather than as a pre-conditioning stimulus.

• The manuscript does not describe how infectivity of different virus isolates was determined before experiments.

We appreciate this observation. We have now clarified that viral infectivity was determined using an immunodetection-based focus-forming assay, which was used to quantify and normalize viral stocks prior to infection experiments. The terminology was also corrected to avoid confusion with classical plaque assays.

• No explanation of how PLPs were distinguished from cell debris or apoptotic bodies during flow cytometry.

We have now expanded the description of the flow cytometry gating strategy. PLPs were identified based on forward and side scatter properties relative to size calibration beads and by CD41 expression, while CD41-negative events and events outside the defined platelet/PLP gate were excluded to minimize inclusion of debris or apoptotic fragments.

• Patient platelet isolation method requires a reference or procedural detail.

The platelet isolation procedure has now been expanded and referenced to established protocols commonly used to minimize platelet activation during flow cytometric analysis. We also added reference 16.

• Number of biological replicates per figure and statistical tests used are not indicated.

We agree and have now clearly indicated the number of independent biological replicates and the statistical tests used for each analysis. This information has been added both to the Statistical Analysis section and to the corresponding figure legends.

• multiple DENV isolates are compared, what normalization strategy was used should be defined. Different isolates may have different infectivity even at the same MOI; this must be addressed.

We appreciate this important point. We have clarified that all viral isolates were normalized by infectious units and used at equivalent MOIs. While isolate-dependent differences in replication kinetics cannot be excluded, normalization by infectious input enabled comparative analysis across experiments. This limitation is now acknowledged in the text.

• Cell viability should have been included as DENV leads to apoptosis.

We acknowledge this limitation. Although formal viability or apoptosis assays were not included in this study, this has now been explicitly stated in the Discussion section. We have tempered our interpretations accordingly and identified this as an important aspect for future studies.

• Indicate number of biological replicates for each experiment.

We agree and have now clearly indicated the number of independent biological replicates and the statistical tests used for each analysis. This information has been added both to the Statistical Analysis section and to the corresponding figure legends.

• Include reference for patient platelet isolation protocol.

The platelet isolation procedure has now been expanded and referenced to established protocols commonly used to minimize platelet activation during flow cytometric analysis. We also added reference 15.

2-In results and figure section
1. Table 1 — Summarize patient demographics (n, sex distribution, severity categories).

We agree with the reviewer. Patient demographics were already included in Table 1; however, to improve clarity, a concise summary of the total number of patients, sex distribution, and clinical severity categories has now been added to the table legend.

2. Figure organization — Reorganize multi-panel figures (e.g., Fig. 1A–F as A with subpanels a–f).

We reorganized Figure 1 to clearly distinguish experimental conditions by using subpanels (a–f) within panel A, as suggested. Other figures already follow a standard quantitative multi-panel format (A–C, D–F) and therefore did not require subpanel restructuring

3. Plaque assay terminology — Antibody-based quantification should be described as “focus-forming assay,” not plaque assay.

We thank the reviewer for pointing this out. The quantification method used in the study was indeed an immunostaining-based assay, and “focus-forming assay (FFA)” is the correct terminology. All references to “plaque assay” have now been replaced with “focus-forming assay” in the Methods, Results, and figure legends for accuracy and consistency.

4. Figure 2 — Add a small legend or color explanation directly near the figure.

We thank the reviewer for this helpful suggestion. A color legend has now been added directly to Figure 2 (panels A and B) to clearly indicate the experimental conditions represented by each color (basal, PMA, DENV1, and PMA + DENV1).

5. Figure 3 — Only performed in K562; please justify why MEG01 was not included.

We thank the reviewer for this important comment. MEG-01 cells were initially included as a complementary megakaryocytic model at a more advanced differentiation stage, and preliminary experiments demonstrated that DENV infection induced changes in CD41 expression that were qualitatively comparable to those observed in K562 cells (Figure 2). However, MEG-01 cultures exhibited higher biological variability across time points and viral isolates, likely reflecting their more committed differentiation state and limited proliferative capacity.

To ensure experimental consistency and allow robust comparative analyses across multiple isolates and infection kinetics, subsequent experiments were therefore conducted using K562 cells as a well-established and stable model of megakaryocyte–erythroid progenitors. Importantly, K562 cells allowed reproducible assessment of isolate-dependent differences in differentiation and infection kinetics, which was the primary objective of Figure 3.

We have clarified this rationale in the revised manuscript.

6. Statistics — Explain why post-hoc analysis was used and ensure consistency across figures.

We thank the reviewer for highlighting the need to clarify the statistical approach. Post-hoc multiple-comparison tests were applied in experiments involving more than two experimental groups, such as comparisons among multiple viral isolates, treatment conditions (basal, PMA, DENV ± PMA), or time points. In these cases, an initial omnibus test was performed (one-way ANOVA or two-way repeated-measures ANOVA, as appropriate), followed by post-hoc testing to identify specific group-to-group differences while controlling for multiple comparisons.

The Methods section has now been revised to explicitly describe the statistical workflow used throughout the manuscript. Specifically, one-way ANOVA followed by Tukey’s post-hoc test was used for comparisons among multiple groups at individual time points; two-way repeated-measures ANOVA with Bonferroni correction was applied to replication-kinetics experiments; unpaired two-tailed Student’s t-tests were used when only two groups were compared; and correlation analyses were performed using Spearman’s rank correlation.

In addition, all figure legends have been carefully reviewed and updated to explicitly state the statistical tests applied, the post-hoc correction used when relevant, and the number of biological replicates.

7. Figure 4 — Include additional methods to assess infection kinetics (viral RNA, infectious viral yield).

We would like to clarify that infection kinetics were indeed assessed in this study and are presented in Figure 1. Specifically, viral replication kinetics in K562 and MEG-01 cells were evaluated by quantification of infectious virus released into culture supernatants using focus-forming assays. These analyses were performed using reference strains of DENV1, DENV4, and ZIKV, as well as one representative clinical isolate from Oaxaca.

The objective of Figure 4, however, was not to further characterize viral replication kinetics, but rather to examine the temporal effects of infection on megakaryocytic differentiation markers and platelet-like particle production following standardized infectious input. For this reason, viral RNA levels or infectious viral yield were not re-evaluated in Figure 4, and infections were normalized by infectious units to enable comparison of downstream cellular responses across conditions.

We have now clarified this distinction between figures in the Results section and have further discussed the scope and limitations of kinetic analyses in the Discussion.

8. Figure 5 — Provide absolute PLP or platelet numbers, not just percentages.

We thank the reviewer for this important observation. Assuming the comment refers to Figure 6, where PLP frequencies are quantified, we agree that presenting absolute PLP or platelet numbers can provide additional information. In our experiments, however, total PLP recovery and cytometer event acquisition varied substantially between replicates and isolates due to differences in culture density, differentiation efficiency, and flow cytometer sampling rate. For this reason, PLP values were normalized and expressed as percentages, which allowed consistent comparison across conditions.

Absolute PLP counts were not consistently available for all replicates and therefore could not be reliably incorporated. We have now clarified in the Results section and in the legend of Figure 6 why percentages were used, and we explicitly acknowledge this as a limitation in the Discussion.

9. Figure 9 — Group patients by severity rather than individual data points; include p-values. Clarify number of healthy controls.

We thank the reviewer for this important suggestion. In the revised version, Figure 9 has been updated to include healthy donors as an explicit comparison group and to display individual data points rather than bar graphs. This approach better reflects inter-individual variability and is more appropriate given the limited sample size.

Accordingly, statistical analyses were revised using non-parametric Mann–Whitney tests. While significant differences were consistently observed between dengue patients and healthy donors, differences between DWW and DWS/SD groups did not reach statistical significance, although a trend toward higher values in the more severe group remained.

The Results and Discussion sections have been revised to reflect these final analyses, and the figure panels presenting redundant bar representations were removed to improve clarity.

Reviewer #2: Alvarado-Silva et al. have evaluated whether infection by various DENV strains, including patient isolates, of megakaryocyte precursors can transfer E protein to platelets. They propose E positive platelets may provide a marker of bone marrow infection. Many of the results are interesting, and this work is generally important for understanding dengue pathogenesis. However, there are numerous areas where the reviewer struggled to understand the figures and results as presented, detailed below.

Major comments:
• Line 74 says 10 blood samples were collected, but 11 patients are listed in table 1.

We thank the reviewer for identifying this inconsistency. This was an error in the text: a total of 11 dengue patient samples were included in the study, as correctly shown in Table 1. Line 74 has been corrected to state that 11 samples were collected. We apologize for the oversight.

• Table 1: It would be helpful to add time since symptom onset when blood was collected as this may impact findings. Additionally, is viremia available for the patients from the time of virus isolation? This would also be helpful to include, if available.

Information on days since symptom onset and quantitative viremia at the time of virus isolation was not consistently available for all patients. We now explicitly state this in the Methods section and acknowledge it as a limitation in the Discussion.

• For Table 3, it would be helpful to include platelet counts, so it is possible to evaluate how severe the thrombocytopenia was.

We thank the reviewer for this suggestion. Platelet counts are now included in Table 3 (reported as ×10⁹/L) to allow assessment of thrombocytopenia severity for each sample. The table legend and the Results section were updated accordingly.

• Line 158: how was it confirmed that the DENV E protein was intracellular and not E protein on the surface? Although intracellular staining was performed, this would stain both surface and intracellular protein.

We appreciate the reviewer’s careful assessment. We agree that fixation and permeabilization-based staining does not definitively discriminate between intracellular and membrane-associated E protein. Importantly, however, the central conclusion of the study does not rely on strict subcellular localization of E protein within platelets, but rather on its origin.

In this regard, we note that primary human platelets exposed ex vivo to DENV under identical staining conditions did not display detectable E positivity. This argues against passive adsorption or direct acquisition of viral antigen by circulating platelets. Instead, our data support a model in which platelet-associated E protein originates during megakaryopoiesis following infection of megakaryocytic precursors, with subsequent transfer to platelet-like particles and circulating platelets.

Whether E protein is retained in intracellular compartments or associated with membrane structures during this process remains to be fully elucidated. We have revised the manuscript to clarify this distinction and to avoid overinterpretation regarding subcellular localization, while emphasizing that the biological relevance of platelet-associated E protein lies in its precursor-derived origin rather than its precise localization.

• Line 236: at day 7 dpi, the CD41 MFI was only significantly higher in DENV1 than PMA, not than basal conditions. The way the sentence is written, it sounds like it was higher across both timepoints. Additionally, PMA + DENV is often lower (blue). It would be helpful if the authors helped explain this point.

We thank the reviewer for this careful observation. We agree that the original wording could be misinterpreted. The text has been revised to clarify that at day 7 post-infection, CD41 MFI was significantly higher in DENV1-infected cells compared with PMA-treated cells, but not relative to basal conditions. We have corrected the sentence to explicitly reflect this comparison.

Regarding the PMA + DENV condition, the reduced CD41 MFI observed at later time points likely reflects the combined effects of pharmacological differentiation and viral infection, which may alter surface marker expression dynamics. We now include a brief explanation in the Results and Discussion to clarify this pattern and avoid overinterpretation.

• Figures 1 and 2: it would be helpful to understand why the authors evaluated the effect of the DENV1 reference strain on MEG-01 but not clinical isolates. It appears clinical isolates were only used on K562 cells.

We thank the reviewer for this comment. MEG-01 cells were used to assess differentiation-associated effects of DENV infection using well-characterized reference strains (DENV1 and DENV4), allowing mechanistic evaluation while minimizing variability associated with isolate-dependent differences in infectivity. These reference strains represent distinct serotypes and provide a controlled framework to examine infection-driven modulation of megakaryocytic differentiation.

In contrast, K562 cells are more permissive to DENV infection and exhibit higher experimental reproducibility, making them more suitable for comparative analyses involving multiple clinical isolates with heterogeneous replication capacities. Accordingly, clinical isolates were evaluated in K562 cells, while MEG-01 cells were used to validate differentiation-related effects using reference viruses. The manuscript has been revised to clarify this rationale.

• For all studies using clinical isolates, it was unclear what MOI was used. Could differences in viral concentrations have contributed to the variable differentiation?

We thank the reviewer for this important point. The Methods section has been revised to explicitly state the multiplicity of infection (MOI) used for each cell model. MEG-01 cells were infected at an MOI of 0.1 using reference strains, whereas K562 cells were infected at an MOI of 1 for all experiments involving clinical isolates. All isolates were normalized by infectious units and used at the same MOI in K562 cells to allow comparative analyses. While isolate-dependent differences in replication efficiency cannot be completely excluded, normalization by MOI minimized variability attributable to differences in viral input.

• Figure 5: the methods say that Pearson correlation was performed, but the figure legend says Spearman’s correlation.

We thank the reviewer for identifying this inconsistency. We have now standardized the correlation analysis and reporting throughout the manuscript to use Spearman’s rank correlation. This approach is more appropriate for the sample size and the given the sample size and potential non-normality of the data. The Methods section and the Figure 5 legend have been corrected accordingly. The overall interpretation is unchanged; notably, no significant correlation is observed at 7 dpi, consistent with the trend previously obtained.

• For all figures where letters are provided instead of statistics comparing groups, it would be much easier of comparisons were presented as in Fig. 2, with bars and stars. It was difficult to follow which analyses were significant.

We thank the reviewer for this helpful suggestion regarding figure clarity. We have revised the figure legends and Results text to more explicitly indicate which group comparisons reached statistical significance. For all figures using letter-based annotations, the figure legends now clearly state that different letters denote statistically distinct groups based on one-way ANOVA with Tukey’s post hoc test (p < 0.05). In addition, the Results section has been revised to explicitly describe significant differences observed at each time point, improving readability and consistency with Figure 2. We also revised the Results and Discussion sections to ensure that interpretations strictly reflect the statistical analyses. Significant differences were primarily observed at 3 dpi, whereas effects at later time points did not consistently reach statistical significance due to increased variability. Accordingly, the text has been modified to avoid overinterpretation of non-significant trends.

• It is very unclear what Fig. 10 is showing. Which in vitro infection was used for this comparison? It appears the authors are referring to the participant’s own clinical isolates, but this isn’t explained anywhere. Additionally, the legend does not seem to match the figure (labeled A and B in the legend, 3 panels in the actual figure) and all the axes have the same labels even though the data seem to be different in each figure.

We thank the reviewer for highlighting the lack of clarity in Figure 10. Upon careful revision, we identified that the confusion resulted from an oversight in the original description of this figure. Figure 10 presents an in vitro correlation between the percentage of E-positive K562 cells and the percentage of CD41⁺E⁺ platelet-like particles generated from the same cultures, and does not involve patient platelet data. The Results text, figure legend, and axis labels have been corrected accordingly to accurately reflect the experimental design and the variables being compared. We also clarified the number of panels and revised the statistical analysis to consistently report Spearman correlation, which is more appropriate for this dataset. These changes resolve the inconsistencies noted by the reviewer and clarify the interpretation of Figure 10.

• K562 cells were used as a model of megakaryocyte-erythroid progenitor cells. K562 cells are commonly used to evaluate antibody-dependent enhancement and are generally not considered infectable without the presence of DENV-specific serum to mediate enhancement. Is this why a high MOI had to be used to achieve infection? Providing some context about the more general use of K562 cells for studying dengue in either the introduction or discussion would be helpful.

We thank the reviewer for this important point regarding the use of K562 cells. While K562 cells are indeed widely used to study antibody-dependent enhancement due to their expression of FcγRII, they have also been reported to support direct DENV infection in the absence of immune serum, we added reference 11. In the present study, all infections were performed without dengue-immune serum, and antibody-dependent enhancement was not assessed. The MOI used was selected based on preliminary titration experiments to ensure reproducible infection and enable comparative analyses across multiple viral isolates, rather than to model ADE. We have revised the Introduction, Methods section and Discussion to provide additional context regarding the use of K562 cells in dengue research and to clarify their role in this study as a model of megakaryocyte–erythroid progenitors under antibody-independent conditions.

Minor comments:
- Orthoflavivirus dengei – dengue?

We thank the reviewer for this comment. The term Orthoflavivirus dengei was used in accordance with the current ICTV taxonomy to denote the official species name. To improve clarity for a broader readership, we have revised the manuscript to introduce both terms at first mention (dengue virus, DENV; species Orthoflavivirus dengei) and subsequently use dengue virus (DENV) throughout the text.

- Fig. 6 and 7 F: label says 4dpi, should it say 7dpi?

We thank the reviewer for noting this. We confirmed that the subpanel F in Figures 6 and 7 corresponds to the 7 dpi time point. The “4 dpi” label was a typographical labeling error, and it has now been corrected to “7 dpi” in the figure panels. No underlying data or analyses were changed.

- Please indicate what orange means in Fig. 9A and B. Dengue with Warning Signs?

We thank the reviewer for pointing this out. Orange symbols correspond to patients with dengue with warning signs or severe dengue (DWS/SD), green symbols indicate dengue without warning signs (DWW), and gray symbols represent healthy donors. This information has been added directly to the figure legend to avoid ambiguity.
