# Supplementary material for: Evidence that dengue virus envelope protein in patient platelets originates from infection of megakaryocytic precursors
Source: PLoS One. 2026 Apr 1;21(4):e0343641. doi: 10.1371/journal.pone.0343641 (PMC13043056; doi:10.1371/journal.pone.0343641)
Supplement: Attachment — Submitted filename: Response_to_Reviewers_auresp_2.docx [file pone.0343641.s002.docx]

Response to the Academic Editor

We thank the Academic Editor for the careful evaluation of our revised manuscript and for the opportunity to further improve it. We have carefully addressed all comments provided by the statistical reviewer and have revised the manuscript accordingly. In particular, we:

Standardized correlation analyses using Spearman’s rank correlation where appropriate.

Clarified statistical methodology throughout the Methods and figure legends.

Revised Figures 5 and 10 to ensure appropriate representation of correlation analyses.

Removed elements that could lead to misinterpretation (e.g., identity reference lines and regression-style presentation).

Ensured full consistency between Results, Discussion, and statistical reporting.

We believe the manuscript has been significantly strengthened and now meets PLOS ONE’s publication criteria.

Response to Reviewers

Manuscript ID: PONE-D-25-49675

Title: Evidence that intracellular dengue virus envelope protein in patient platelets originates from infection of megakaryocytic precursors

Authors: Alvarado-Silva et al.

Journal: PLOS ONE

Reviewer #3: In this study the investigators investigated whether infection of megakaryocytic precursors contributes to the generation of platelets carrying dengue virus proteins. It appears that the statistical issues have been addressed, the one dealing with the analysis of multiple groups with post hoc comparisons has certainly been clarified. Reading the manuscript and track changes a number of clarifications and additions have been addressed as well. The revision is much improved. There are a few minor clarifications needed:

1. On Figure 5 what is the 45 degree line? Please explain that to the reader. Also the analysis looks like a method comparison approach. This is not necessarily handled with a correlation. One should consider a Bland Altman type of analysis as well.

Response:

We appreciate this important observation. The 45-degree reference line has been removed from Figure 5 in the revised manuscript to avoid any suggestion of a method-comparison or agreement analysis.

Figure 5 evaluates biological association between two distinct biological variables (percentage of infected cells and differentiation marker expression), not agreement between measurement methods. Therefore, a Bland–Altman analysis was not appropriate for this context.

We have clarified in both the figure legend and the Results section that each data point represents one independent infection condition (one viral isolate), plotted as the mean of biological replicates.

We have revised the figure and legend accordingly to clarify the nature of the analysis.

2. On Figure 10 why the wide error bars on some of the points? Since this is apparently a regression the R squared is usually reported and not only the r as the authors have done.

Response:

We thank the reviewer for this comment. In the revised version, error bars have been removed from Figure 10. The correlation analysis is now performed using one point per independent infection condition (mean of biological replicates), consistent with the experimental design.

Furthermore, we standardized the analysis using Spearman’s rank correlation coefficient. Because Spearman correlation does not model linear regression, R² is not applicable. Therefore, we report Spearman’s r and the corresponding two-tailed p value in the figure panels.

The Methods, Results, and figure legends have been revised accordingly to ensure clarity and consistency.

3:Since the sample size is so small, then for some of the analyses, why not the non parametric Kruskal Wallis approach for a multiple group comparison and the Friedman test for repeated measures?

Response:

We appreciate this suggestion. For correlation analyses (Figs 5 and 10), we now uniformly apply Spearman’s rank correlation, which does not assume normal distribution and is appropriate for small sample sizes.

For multi-group comparisons involving flow cytometry data, one-way ANOVA with Tukey’s post hoc test was retained because the data met parametric assumptions based on distribution assessment and homogeneity of variance within experimental replicates.

For replication kinetics experiments, two-way repeated-measures ANOVA was employed to model both viral strain and time as factors, including their interaction. The Friedman test does not accommodate factorial designs with interaction terms; therefore, two-way repeated-measures ANOVA was considered more appropriate for these experiments.

These statistical approaches are now explicitly justified in the revised Methods section.

4: In Figure 10 since the two active groups are being compared to the control a Dunnet’s test might be considered.

Response:

Figure 10 presents correlation analyses between two continuous variables (precursor infection and PLP-associated E protein levels) rather than group-wise comparisons against a single control group. Therefore, Dunnett’s multiple-comparison test is not applicable in this context.

This has been clarified in the revised figure legend and Results section.

Additional Clarifications

As part of this revision, we also:

Standardized correlation analyses in both Figures 5 and 10 using Spearman’s rank correlation.

Removed any reference to regression modeling or R².

Revised the Discussion to avoid overinterpretation of non-significant correlations at later time points.

Ensured consistency between statistical methods, figure legends, and Results descriptions.

We are grateful for the reviewer’s detailed statistical feedback, which has strengthened the rigor and clarity of our manuscript.
